# Genome-Wide Analysis of MYB10 Transcription Factor in *Fragaria* and Identification of QTLs Associated with Fruit Color in Octoploid Strawberry

**DOI:** 10.3390/ijms222212587

**Published:** 2021-11-22

**Authors:** Abinaya Manivannan, Koeun Han, Sun Yi Lee, Hye-Eun Lee, Jong Pil Hong, Jinhee Kim, Ye-Rin Lee, Eun Su Lee, Do-Sun Kim

**Affiliations:** Vegetable Research Division, National Institute of Horticultural and Herbal Science, Rural Development Administration, Wanju 55365, Korea; abinayamanivannan@gmail.com (A.M.); hke1221@korea.kr (K.H.); sylee2@korea.kr (S.Y.L.); helee72@korea.kr (H.-E.L.); hjp3467@korea.kr (J.P.H.); sayzinni@korea.kr (J.K.); lyr1219@korea.kr (Y.-R.L.); lus4434@korea.kr (E.S.L.)

**Keywords:** *Fragaria*, MYB10, fruit color, quantitative trait locus (QTL)

## Abstract

The genus *Fragaria* encompass fruits with diverse colors influenced by the distribution and accumulation of anthocyanin. Particularly, the fruit colors of strawberries with different ploidy levels are determined by expression and natural variations in the vital structural and regulatory genes involved in the anthocyanin pathway. Among the regulatory genes, MYB10 transcription factor is crucial for the expression of structural genes in the anthocyanin pathway. In the present study, we performed a genome wide investigation of MYB10 in the diploid and octoploid *Fragaria* species. Further, we identified seven quantitative trait loci (QTLs) associated with fruit color in octoploid strawberry. In addition, we predicted 20 candidate genes primarily influencing the fruit color based on the QTL results and transcriptome analysis of fruit skin and flesh tissues of light pink, red, and dark red strawberries. Moreover, the computational and transcriptome analysis of MYB10 in octoploid strawberry suggests that the difference in fruit colors could be predominantly influenced by the expression of MYB10 from the *F. iinumae* subgenome. The outcomes of the present endeavor will provide a platform for the understanding and tailoring of anthocyanin pathway in strawberry for the production of fruits with aesthetic colors.

## 1. Introduction

The gene expression at transcription level is crucial for numerous physiological and biochemical processes in the biological organism. The regulation of gene transcription is determined by various factors, among which, transcription factors are predominantly important. Plant transcription factors influence the regulation of target genes that are involved in growth, development, and response to environmental stresses in plants [1]. In general, transcription factors are proteins distinguished into several families based on the structure of the DNA binding domains [2]. Transcription factors function by binding to the sequence specific cis-acting promoters to enhance or suppress the regulation of target genes. MYB is one of the important transcription factors involved in the regulation of various vital genes involved in diverse processes particularly in anthocyanin biosynthesis in plants.

In horticultural crops, biosynthesis of anthocyanin and its accumulation in tissues like flowers and fruits is important for breeding new varieties. In strawberry, the ripeness and esthetic quality of fruits are predominantly determined by the accumulation of anthocyanin [3]. Apart from the esthetic feature, anthocyanin pigments render protection against various biotic and abiotic stresses in plants [4]. The red, purple, violet, and pink pigments in fruits and flowers are due to the compounds formed from the phenylpropanoid pathway such as hydroxycinnamic acid, isoflavones, flavonols, phlobaphenes, and pro-anthocyanidins [5]. The phenylpropanoid pathway consists of several vital enzymes that are encoded by the early and late anthocyanin biosynthesis group genes such as *phenylalanine ammonia lyase* (*PAL*), *chalcone hydrolase isomerase* (*CHI*), and *dihydro flavanol reductase* (*DFR*) [6]. These structural genes are influenced by the regulatory genes mainly by MYB transcription factors directly or by co-regulation with other transcription factors like bHLH and WD40 repeats by forming a MBW complex [7].

In recent years, there has been an increasing progress in the understanding of molecular mechanisms of pigmentation in fruit and flowers through multiple investigations of transcriptional regulation of key genes such as MYB transcription factors [8]. The MYB transcription factor belongs to R2R3 MYB transcription factors which play vital regulatory roles in determining the accumulation of anthocyanin in several horticultural crops including strawberry [9]. MYB transcription factors can be classified into three subfamilies based on the number of highly conserved imperfect repeats in the DNA-binding domain including R3 MYB (MYB1R) with one repeat, R2R3 MYB with two repeats, and R1R2R3 MYB (MYB3R) with three repeats [8]. Among these MYB transcription factors, R2R3-MYBs constitute the largest transcription factor gene family in plants, with more than 126 R2R3 MYB genes identified in *Arabidopsis* [8]. In strawberry, FaMYB10 expression caused the synthesis of high levels of anthocyanin [9]. Even though studies have identified the importance of MYB10, the genome level understanding of the MYB10 in *Fragaria* is still under investigation. A recent report by Castillejo et al. [10] demonstrated the allelic variation in MYB10 acts as an important factor that influences the fruit color in strawberry. The determination of genomic loci or genes controlling the variation in fruit color in octoploid strawberry is important for the breeding of new strawberry cultivars with desired esthetic fruit colors. However, the complex genome and ploidy level in cultivated strawberry pose difficulty for the investigation of genomic loci or mapping of quantitative trait loci associated with fruit colors. The recent advancements in next generation sequencing enabled the availability of the whole genome sequence of diploid and octoploid strawberry. The reference genomes of heterozygous ‘Camarosa’ [11] and homozygous ‘Wongyo 3115’ [12] cultivated strawberries can facilitate the understating of gene regulation and development of markers associated with fruit colors in octoploid strawberry.

Multiple studies were conducted to figure out the locus controlling fruit colors evaluated by visual evaluation score, color parameters (L, a, b), and anthocyanin contents [10,13,14,15,16,17]. Total anthocyanin contents of strawberry fruits were correlated with values measured by colorimeter or the metabolites including pelagonidin derivatives. In addition, QTLs were detected to control color-related traits commonly in multiple chromosomal regions. By comparison with the physical map of *F*. *vesca* and *F*. × *ananassa*, *MYB10*, and *flavonoid 3′-hydroxylase* (*F3’H*) were suggested as candidate genes for fruit color [10,16]. *F3’H*, a structural gene involved in flavonoid biosynthesis, showed significant correlation with flavonoid levels in F_1_ lines [16]. Although, multiple QTLs were identified, possible candidate genes could be identified from genome-based QTL analysis.

The molecular insight on MYB10 can be of vital knowledge for tailoring the anthocyanin biosynthesis in strawberry. Therefore, in the present study, we have identified the *MYB10* genes present in five diploids and two octoploid strawberry genomes. The transcriptome analysis of fruits from four different strawberry cultivars with different fruit colors ranging from light pink to dark red were utilized for the expression analysis of structural and regulatory genes involved in anthocyanin biosynthesis. In addition, the six QTLs associated with fruit color were detected and utilized for the prediction of candidate genes controlling the skin and flesh colors in strawberry. Overall, the outcomes of the present study can benefit the breeding of strawberry cultivars with varying fruit colors and also facilitate the development of trait specific markers for strawberry breeding.

## 2. Results

### 2.1. Characterization of MYB10 in Diploid and Octoploid Species of Fragaria

A total of 14 *MYB10* genes were identified in eight genomes of *Fragaria* (Table 1). All the identified *MYB10* genes were located on the chromosome 1. The diploid genomes consisted of one *MYB10* gene, whereas the octoploids consisted of four. The octoploid cultivated strawberry ‘Wongyo 3115’ consisted of four homeologs of *MYB10* located in each subgenome, whereas ‘Camarosa’ consisted one *MYB10* homeolog in each Ch1-1, Ch1-2 and Ch1-3 encompassed two homeologs of *MYB10,* whereas no *MYB10* was identified in Ch1-4. In homozygous octoploid strawberry, two *MYB10* (*g00115370* and *g00119641*) were annotated as *MYB113* in the ‘Wongyo 3115’ annotation file. The gene size of *MYB10* ranged from 1.8 to 3.2 kb in diploids and 0.4 to 11.1 kb in octoploid strawberry. Among the diploids, *F*. *nilgerensis* consisted of the largest *MYB10* with 3.2 kb and *F*. *iinumae* encompassed the smallest *MYB10* with gene size of 1.8 kb. In homozygous octoploid strawberry ‘Wongyo 3115’, the smallest *MYB10* homeolog *g00101712* comprised of 467 bp, whereas the other *MYB10* homeologs were of similar size to the diploids. Similarly, in heterozygous octoploid strawberry ‘Camarosa’ the *MYB10* homeolog *Fvb1-3-augustus-gene-143.29* (*143.29*) consisted of 331 bp whereas the size of *Fvb1-2-snap-gene-157.15* (*157.15*) *MYB10* homeolog consisted of 11.1 kb.

### 2.2. Structure and Phylogeny of MYB10 in Diploid and Octoploid Species of Fragaria

**Figure 1:**
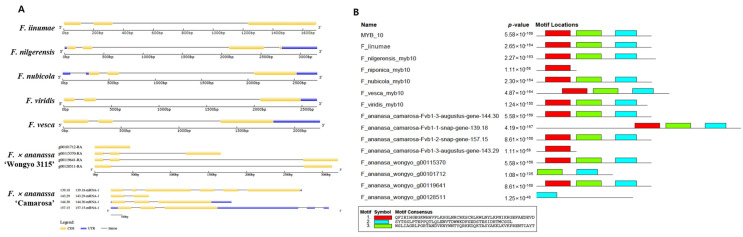
Structure of *MYB10* in diploid and octoploid *Fragaria* (**A**) and motifs identified in *Fragaria MYB10* (**B**).

After the identification of *MYB10* in diploid and octoploid *Fragaria* species, the gene structure and motifs were analyzed (Figure 1). Gene structure of *MYB10* consisted of three exons separated by two introns. All the diploids consisted of three exons and two introns. However, in homozygous octoploid *F*. × *ananassa* ‘Wongyo 3115’ two *MYB10* (*g00115370* and *g00119641*) homeologs encompassed similar exons and introns like diploids. The *g00101712* displayed only one exon and *g00128511* possessed one intron and two exons. In the heterozygous octoploid strawberry *F*. × *ananassa* ‘Camarosa’ the two homeologs *maker-Fvb1-3-augustus-gene-144.30* (*144.30*) and *157.15* displayed similarity with the diploids, whereas two exons and one s*nap_masked-Fvb1-3-processed-gene-138.18* (*139.18*) intron were identified in *F*. × *ananassa* ‘Camarosa’ *143.29*. On the other hand, six exons spaced by five introns were identified in the homeolog *139.18*. Further, motif analysis using the MEME tool demonstrated the occurrence of three conserved motifs in most of the MYB10 proteins of *Fragaria* except in *F*. *nipponica*, *F*. × *ananassa* ‘Wongyo 3115’ (*g00128511* and *g00101712*), and *F*. × *ananassa* ‘Camarosa’ (*143.29*).

**Figure 2:**
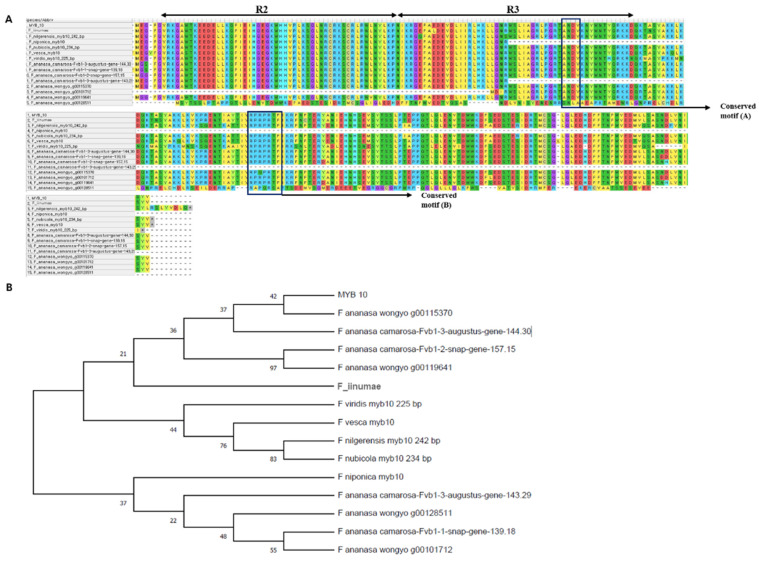
Multiple protein sequence alignment of *Fragaria* MYB10: (**A**) the conserved motif A [ANDV] and motif B [RPRPRTF] are highlighted; the phylogenetic relationship between MYB10 transcription factors in *Fragaria* (**B**).

The multiple sequence alignment of MYB10 proteins suggested the presence of signature R2 and R3 domains in *Fragaria* (Figure 2A). However, the R2 domain and partial R3 domains were absent in *F*. × *ananassa* ‘Wongyo 3115’ (*g00101712*). In addition, two conserved motifs A [ANDV] and B [RPRPRTF] required for the activity of MYB10 was identified. According to previous report, the presence of motif A is vital for the interaction with bHLH co-factors for the promotion of anthocyanin biosynthesis [18], whereas the C-terminal conserved motif B is important for regulation of anthocyanin genes [18]. The conserved motif A and B were absent in *F*. *nubicola*, *F*. × *ananassa* ‘Camarosa’ (*143.29*), and *F*. × *ananassa* ‘Wongyo 3115’ (*g00128511*) MYB10 transcription factors. The first arginine residue was substituted by lysine in motif B of *F*. × *ananassa* ‘Wongyo 3115’ (*g00101712*) and the third arginine residue was replaced by glutamine in the *g00115370*. Further, to investigate the phylogenetic relationship among *Fragaria* species, a dendrogram was constructed for the amino acid sequence of MYB10 using maximum likelihood algorithm (Figure 2B). The tree displayed two major clusters with most of the diploids such as *F*. *iinumae*, *F*. *vesca*, *F*. *viridis*, *F*. *nilgerensis*, and *F*. *nubicola* along with the octoploid *F*. × *ananassa* ‘Camarosa’ (*144.30* and *157.15*) and *F*. × *ananassa* ‘Wongyo 3115’ (*g00115370* and *g00119641*) grouped in cluster 1. Furthermore, cluster 2 consisted of diploid *F*. *nipponica*, octoploids *F*. × *ananassa* ‘Camarosa’ (*143.29* and *139.18*), and *F*. × *ananassa* ‘Wongyo 3115’ (*g00128511* and *g00101712*). The clusters 1 and 2 are further divided into five and four sub-groups, respectively. MYB10 from *F*. *iinumae* displayed a close relationship with *F*. × *ananassa* ‘Wongyo 3115’ (*g00119641*) and *F*. × *ananassa* ‘Camarosa’ (*157.15*). Other diploids, such as *F*. *vesca*, *F*. *viridis*, *F*. *nilgerensis*, and *F*. *nubicola,* exhibited a close evolutionary relationship.

### 2.3. RNA-Seq Based Expression Profiling of MYB10 and Anthocyanin Biosynthesis Genes

**Figure 3:**
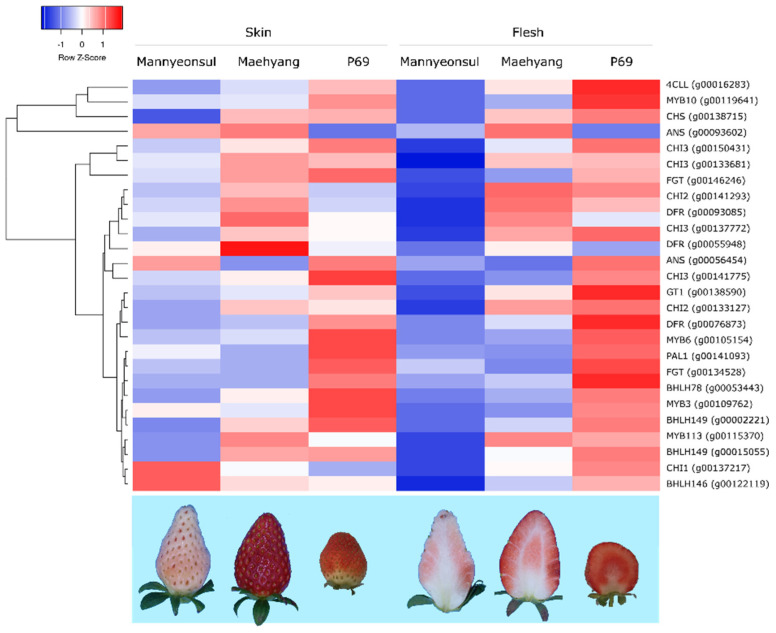
Heatmap displaying the differentially expressed regulatory and structural genes (fold-change > 2 and FDR < 0.05) involved in the anthocyanin pathway in skin and flesh of light pink and red strawberry varieties in different developmental stages.

The differential expression profile of 28 important genes including both structural and regulatory genes involved in anthocyanin biosynthesis in the skin and flesh tissues of three strawberry cultivars were investigated (Figure 3, Table S1). The three cultivar ‘Mannyeonsul’, ‘Maehyang’, and ‘P69’ had light pink, red, and dark red fruit color and showed difference in L* (brightness) and a* (redness) value (Figure 3, Table S2). The *MYB10* homeolog from Ch1-2 (*g00119641*) contributed by *F*. *iinumae* subgenome of octoploid strawberry displayed higher relative expression levels, whereas mere expression of Ch1-3 (*g00115370*) was observed in the transcriptome analysis. However, no significant expression of the other *MYB10* genes were identified. The transcriptome results suggested the higher expression of most of the structural genes, such as *PAL* (*g00141093*), *glycosyltransferase* (*GT*; *g00138590*), *flavonoid glucosyltransferase* (*FGT*; *g00134528*), *4-coumaroyl-CoA ligase-like* (*4CLL*; *g00016283*), *CHI3* (*g00150431*), *CHI2* (*g00133127*), and *chalcone synthase* (*CHS*; *g00138715*), were observed in the skin and flesh tissues of dark red ‘P69’. On the other hand, the light pink ‘Mannyeonsul’ displayed a lower expression of the vital structural genes involved in anthocyanin biosynthesis. The higher transcript levels of *DFR* (*g00076873*, *g00055948*, and *g00093085*) and *anthocyanidin synthase* (*ANS*; *g00056454*) were identified in the skin tissue of red fruit bearing ‘Maehyang’ cultivar. Further, increase in the abundance of the *MYB10* transcript (*g00119641*) was observed in skin and flesh tissues of ‘P69’ while *MYB10* displayed lower expression in the light pink fruits of the ‘Mannyeonsul’ cultivar. The expression of *bHLH* transcription factor *bHLH149* (*g00015055* and *g00002221*) and *bHLH146* (*g00122119*) were higher in both the tissues of ‘P69’ and lower in ‘Mannyeonsul’. However, the *bHLH78* (*g00053443*) expression was least in skin and flesh tissues of ‘Maehyang’ cultivar.

### 2.4. Relative Expression Analysis of MYB10 and Anthocyanin Biosynthesis Genes

**Figure 4:**
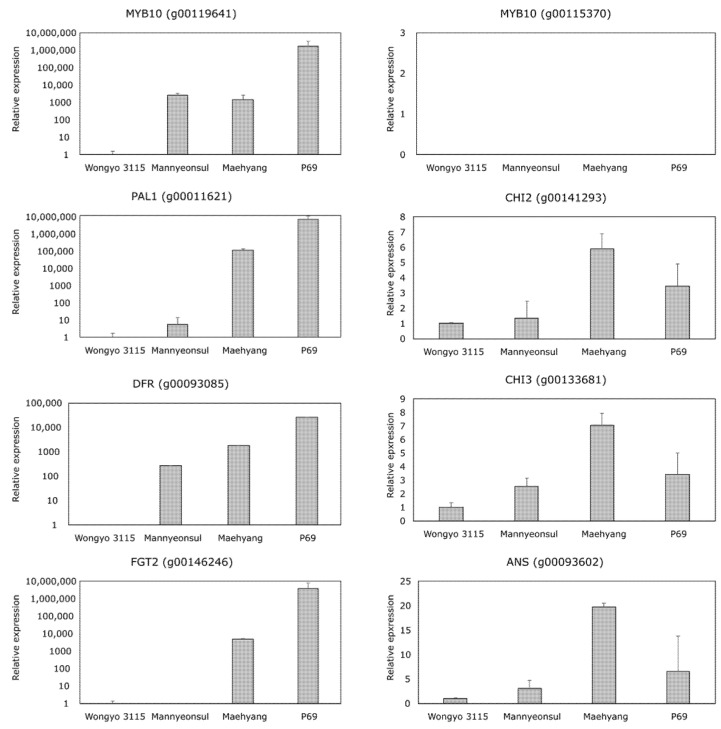
Relative expression analysis of the vital genes involved in the anthocyanin pathway using real-time PCR. The values are relative expression level compared to ‘Wongyo 3115’ and standard deviation.

In order to gain insight into the MYB10 mediated regulation of anthocyanin biosynthesis, gene expression in mature flesh tissue of four cultivars with varying fruit colors was investigated (Figure 4). In a similar pattern with the RNA-seq results, the structural genes *PAL1* (*g00011621*), *CHI2* (*g00141293*), *CHI3* (*g00133681*), *DFR* (*g00093085*), *FGT2* (*g00146246*), and *ANS* (*g00093602*) showed higher expression in ‘P69’ and ‘Maehyang’ compared to ‘Wongyo 3115’, which has a white flesh color. Similarly, the expression level of the homeolog *MYB10* (*g00119641*) showed correlation with the redness of the fruit flesh, whereas *MYB10* (*g00115370*) was not expressed in all cultivars.

### 2.5. Identification of QTLs Associated with Fruit Color

**Figure 5:**
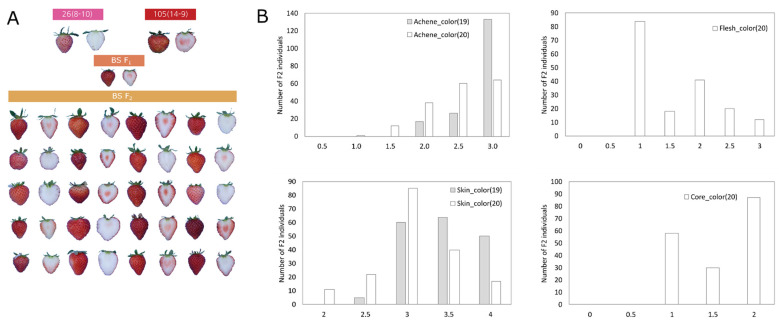
(**A**) Phenotype of ‘BS F_2_ (II)’ fruits utilized for the fruit color QTL identification evaluated for 2019 and 2020. (**B**) Distribution of fruit color related traits in ‘BS F_2_ (II)’.

For the QTL analysis associated with the fruit color trait, the phenotyping was performed for two consecutive years (2019–2020). The skin and achene colors were evaluated for the year 2019, while flesh and core color were evaluated additionally for 2020. The maternal line displayed pink skin with white flesh and core color. The paternal line showed red skin with light red flesh and red core color (Figure 5A; Supplementary Dataset S2). Fruit skin color exhibited normal distribution in F_2_ progenies, while achene color and flesh color biased to red and white, respectively (Figure 5B). QTLs controlling fruit color were analyzed using bin map of ‘BS F_2_ (II)’ [12] and the color-related traits evaluated for 2019 and 2020. QTLs detected on the common physical position in ‘Wongyo 3115’ genome were combined to one. A total of seven combined QTLs were detected on chromosomes 1-2, 1-4, 2-1, 6-4 and 7-4 (Table 2). *Col_1-2* detected on chromosome 1-2 and could explain all fruit color-related traits with 10-76% of phenotypic variation. *Col_6-4_2* was identified to control skin color for 2019 and achene color for 2020. Other QTLs were only detected for one trait evaluated in one year.

### 2.6. Identification of Predicted Candidate Genes Associated with Fruit Color Using QTL and RNA-Seq Analysis

The QTL information and ‘Wongyo 3115’ annotation data were employed for the prediction of candidate genes associated with fruit color. To investigate the candidate genes associated with fruit color, genes that displayed differential expression between matured fruits of pink color ‘Mannyeonsul’, and red color ‘Maehyang’, and ‘P69’ located in QTL regions were selected. The QTL regions covered a total of 1605 differentially expressed genes based on the physical position details retrieved from the ‘Wongyo 3115’ annotation data (Table S3). Among the QTL regions, *Col_6-4* consisted of a higher number of genes (686) followed by *Col_2-1* (281 genes).

**Figure 6:**
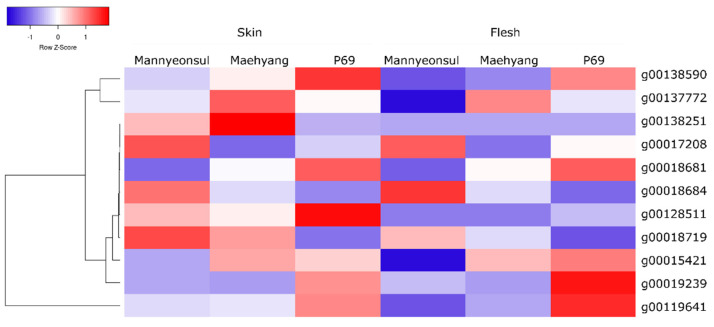
Heat map representation of 11 differentially expressed predicted candidate genes (fold-change > 2 and FDR < 0.05) associated with fruit color in three cultivars of strawberry.

The paired analysis between two inbred lines was performed with the filtering criteria of two-fold-change difference. The number of differentially expressed genes in only skin and flesh were 386 and 394, respectively. In contrast, a total of 825 genes showed a difference in the expression in both tissues. Further, the differentially expressed genes located in the QTL regions were extracted based on the physical position from the annotation data of the ‘Wongyo 3115’ assembly. A total of 11 potential candidate genes including vital structural and regulatory genes associated with fruit color were identified by utilizing the transcriptome and QTL data (Table 3; Figure 6, Table S4). In particular, we identified the *DFR*, *ANS*, *CHI3*, *GT1*, and *MYB10* transcription factors in the QTL regions, which play a vital role in the determination of fruit color in strawberries. In addition, transcriptome analysis indicated the up-regulation of structural and regulatory genes in red fruit bearing ‘P69’ cultivar than the light pink fruits bearing the ‘Mannyeonsul’ cultivar. In addition, the QTL regions consisted of several regulatory genes such as *MYB* (4), *bHLH* (4), and *WD-repeats* (4) with potential roles in anthocyanin pathway (Table S3).

## 3. Discussion

Strawberry is a widely cultivated horticultural crop with tremendous nutritional and commercial importance. The aesthetic and nutraceutical values of strawberry renders several benefits to human health [3]. The fruit colors of strawberry are determined by the distribution and accumulation of water-soluble, flavonoid based pigments called anthocyanin. Based on the anthocyanin contents the strawberry fruits are observed in different colors from white to dark red. The anthocyanin levels significantly increase during the development of fruits and reaches higher levels at the ripening stage [19]. Recent reports suggested the predominant role of MYB10 transcription factor in the determination of fruit color in strawberry [9,10,20]. Therefore, in the present endeavor, we have performed genome-wide investigation of *MYB10* transcription factor in diploid and octoploid strawberry.

For the genome-wide analysis, a total of eight genomes including six diploids and two octoploids were investigated. A sequence-based homology search using the *F*. × *ananassa MYB10* resulted in the 14 *MYB10* genes in the genomes. All the diploids consisted of one *MYB10,* whereas the octoploids possessed four homeologs of *MYB10*. In all the genomes, the *MYB10* loci were observed in chromosome 1. In general, the gene structure of *MYB10* encompassed three exons and two introns [10]. In *Fragaria*, all diploids consisted of three exons separated by two introns whereas in octoploids the *MYB10* homeologs *g00128511* and *143.29* displayed two exons and one intron. On the other hand, the homeolog in *F*. × *ananassa* ‘Camarosa’ *139.18* possessed six exons spaced by five introns and *F*. × *ananassa* ‘Wongyo 3115’ *g00101712* displayed only one exon. Duplication events in the genome of octoploid strawberry resulted in four homeologs *MYB10* in the homozygous ‘Wongyo 3115’ genome. *MYB10* was identified in each subgenomes; however, in the heterozygous ‘Camarosa’, Ch1-3 consisted of two *MYB10* homologs and none was identified on Ch1-4. Genome doubling and duplication events in the genome of octoploid strawberry led to the occurrence of multiple *MYB10* homeologs in comparison with diploids.

Despite the presence of multiple homeologs, the *MYB10* homeologs in ‘Wongyo 3115’ (*g00119641* and *g00115370*) displayed significant expression in the strawberry fruits employed in this study. In addition, *MYB10* (*g00119641*) contributed by *F*. *iinumae* subgenome of octoploid strawberry displayed higher expression in comparison with *g00115370*. According to Fu et al. [21], the homeologous gene expression in allopolyploid crops exhibit spatial, temporal, and complex features that also tend to result from alternative splicing. The differential gene expression in polyploid results in functional plasticity and incorporates novel phenotypes that permit the adaptability of polyploid more efficiently to environmental fluctuations [21]. Moreover, our results are in accordance with the recent report [10] suggesting the anthocyanin biosynthesis regulation is mediated by the *MYB10* homeolog contributed by the *F*. *iinumae* subgenome of octoploid strawberry. The disruption of MYB10 function could be attributed to the difference in the gene structure, occurrence of repeats in the functional regions, and premature codon displacement; these factors could have led to the mere expression or non-expression of other *MYB10* homeologs in octoploid strawberry.

Anthocyanin biosynthesis and accumulation in plants are determined by the phenylpropanoid pathway. The expression of major structural genes such as *PAL*, *GT*, *FGT*, *4CLL*, *CHI,* and *CHS* in synergistic expression correlation with regulatory genes like *MYB*, *bHLH*, and *WD-40* repeats facilitate the phenotypic variations in the anthocyanin distribution in strawberry [10]. Therefore, to investigate the expression of vital structural and regulatory genes involved in anthocyanin biosynthesis, the transcriptome analysis in three cultivars of strawberry such as ‘Mannyeonsul’ (light pink fruits), ‘Maehyang’ (red fruits), and ‘P69’ (dark red fruits) were conducted. The results revealed a higher expression of *PAL* (*g00141093*), *GT* (*g00138590*), *FGT* (*g00134528*), *4CLL* (*g00016283*), *CHI3* (*g00150431*), *CHI2* (*g00133127*), and *CHS* (*g00138715*) in the dark red fruit bearing ‘P69’ cultivar and lower expression in light pink fruits of ‘Mannyeonsul’. Similarly, the transcriptome levels of *MYB10* correlated with expression of structural genes. *MYB10* transcription factor acts as one of the important factors that influence anthocyanin biosynthesis pathway in both diploid *F*. *vesca* [22] and octoploid *F*. × *ananassa* [10]. According to Lin-Wang et al. [23], the RNAi based silencing of *MYB10* correlated with the decreased in the expression levels vital genes such as *CHI*, *CHS*, *F3H*, and *DFR* responsible for anthocyanin synthesis. Nevertheless, Lin et al. [24] reported that the MYB10 gene displayed non-significant difference in expression between red and white fleshed strawberry. In addition, Medina-Puche et al. [25] evidenced that the gene *ANS* is not regulated by transient silencing of FaMYB10 in cultivated strawberry. These studies suggest that the expression of MYB10 acts as an additional factor in the regulation of vital structural genes involved in the anthocyanin biosynthesis pathway in strawberry. Moreover, MYB10 also influenced the expression of other regulatory genes such as *bHLH33*, *MYB89*, and *MYB1* [23]. Similarly, the relative expression of the structural genes displayed a positive relation with the expression of *MYB10* (*g00119641*).

Consequently, the RNA-seq data confirmed the positive regulation of MYB10 transcription factor and the corresponding structural genes in the dark red matured fruits of ‘P69’. The stage specific relative expression analysis using qPCR suggested that the expression of *MYB10* as well as the vital structural genes are increased during the ripening stage in all the cultivars. However, a higher fold increase has been observed in the skin and flesh tissues of dark red fruit bearing ‘P69’ cultivar in comparison with other cultivars. Several reports have evidenced the positive relationship of the *MYB10* regulatory gene with structural genes involved in early and late anthocyanin biosynthesis in various crops such as apple [26], pear [27], sweet cherry [28], and peach [29]. Recently, Wang et al. [30], reported an 8-bp insertion in the *MYB10* coding region which correlated with the color loss in octoploid strawberry cultivar ‘Snow Princess’. This study was further evidenced by Castillejo et al. [10], illustrating the allelic variation in the *MYB10* determine the color variation in skin and flesh tissues of strawberry. Similarly, in *F*. *vesca* ‘Yellow Wonder’, a single nucleotide mutation in the 35th position of *MYB10* sequence encoding the R2 DNA binding domain resulted in yellow color fruits [22].

In this QTL study, seven QTLs controlling fruit colors were detected (Table 3) and two QTLs (*Col_1-2* and *Col_6-4_2*) control multiple traits. For QTL *Col_1-2*, F*_2_* individuals with maternal genotype showed darker skin, flesh, and core color than those of paternal genotype. Conversely, paternal genotyped individuals showed darker achene color. *Col_6-4_2* also showed dissimilar pattern for skin and achene color. In a similar manner, achene color was negatively correlated with other fruit color related traits whereas the other traits were positively correlated each other (Table S5). ‘8-10’ and ‘Mannyeonsul’ showed pink skin and red achene while ‘105 (14-9)’ and ‘P69’ showed dark red skin and yellowish achene. In previous research, hormone accumulation and gene expression related to fruit development showed inconsistency in achene and receptacle [31,32]. From these findings, we assume that achene development and ripening might be controlled by different a mechanism from the receptacle. *MYB10* was proposed as a strong candidate for achene, skin, flesh, and core color of fruits. QTLs controlling skin color parameter a and b detected on LGIa [13], and parameter a detected on LG1.3 [17], were also located on Chr1-2 linked to the *MYB10* gene. Gene expression analysis support the MYB10 mediated regulation of structural genes which influence the accumulation of anthocyanin in fruit tissues of octoploid strawberry cultivars ‘Mannyeonsul’, ‘Maehyang’, and ‘P69’.

The ‘Wongyo 3115’ annotation information and QTL information also facilitated the prediction of potential candidate genes that could influence the skin and flesh colors in octoploid strawberries. These candidate genes participate in several essential processes such as sugar metabolism, secondary metabolism, auxin metabolism, hormone biosynthesis, and DNA binding. Potential genes such as *DFR*, *ANS1*, *CHI3*, *GT1*, *UGT*, and *MYB10* with an active role in anthocyanin biosynthesis have been identified as candidate genes in *Col_6-4_1*, *Col_6-4_2*, *Col_7-4*, and *Col_1-2* QTL regions.

The identification of candidate genes influencing the anthocyanin pathway can enhance the tailoring of the pathway to acquire strawberry fruits with varying colors. The importance of the candidate genes such as *DFR*, *ANS1*, *CHI3*, *GT1*, *UGT*, and *MYB10* in the biosynthesis of anthocyanin have been evidenced in several horticultural crops. Particularly, in cultivated strawberry, the fruit color is vital for the consumer preference and therefore considered as a vital trait for strawberry breeding. Similarly, we have identified transcription factors belonging to MYB, bHLH, and WD families in the QTL regions. Several researchers have asserted the important functions of transcription factors and hormones in strawberry fruit development [31,33,34]. In future endeavors, the vital role of TFs associated with the fruit color can be attained and the present QTL data can facilitate the process. Overall, the outcomes of the present study provide a valuable resource for future functional characterization and development of trait specific markers associated with MYB10 for breeding new cultivars of strawberry with diverse fruit color.

## 4. Materials and Methods

### 4.1. Plant Materials

To analyze the gene expression related to fruit color, two varieties ‘Maehyang’ and ‘Mannyeonsul’, and one inbred line ‘P69’, were used. ‘Maehyang’ originated from a cross between ‘Tochinomine’ and ‘Akihime’ and has red skin, flesh, and core color (registration number 5160, Korea Seed and Variety Service). ‘Mannyeonsul’ is known as mutant of ‘Akihime’ and shows pink skin and flesh color (registration number 7867, Korea Seed and Variety Service). ‘P69’ is an inbred line derived from ‘Benihoppe’ by nine self-pollination and has red skin, flesh, and core color. A total of 186 ‘BS F_2_ (II)’ population derived from a cross between ‘8-10’ and ‘105 (14-9)’ were used for QTL analysis. All plants were grown in raised beds located in Wanju, Republic of Korea. ‘BS F_2_ (II)’ was grown for two consecutive years (2019 and 2020) and four plants per F_2_ line were planted for phenotyping.

### 4.2. Identification of MYB10 Transcription Factors in Fragaria

The genomic resources of six diploid *Fragaria* species and octoploid *F*. × *annanasa* ‘Camarosa’ were downloaded from the GDR database (https://www.rosaceae.org/species/fragaria/all (accessed on 2 June 2021)) and *F*. × *annanasa* ‘Wongyo 3115’ from GenBank (https://www.ncbi.nlm.nih.gov/assembly/GCA_019022445.1 (accessed on 25 June 2021)). For the identification of *MYB10* transcription factor, homology sequence search using BLAST tool, with the query *MYB10* transcription factor (QIZ03070) from *F*. × *ananassa,* was employed. The sequences with 100 to 98% similarity were selected for further analysis. The location of *MYB10* sequences were extracted from the genome feature file (gff) for each genome. Multiple sequence alignments of the MYB10 protein sequences were performed using Clustal X (version 2.1) in multiple alignment mode. A maximum likelihood phylogenetic tree was constructed using MEGA 6.0 with the following parameters: 1000 bootstrap replicates, pairwise alignment, Dayhoff model, Gamma distributed (G), 2 Gamma parameters, and pairwise deletion.

### 4.3. Analysis of Conserved Motifs, Gene Structures, and Cis-Elements

The MYB10 protein sequences were analyzed with the MEME (https://meme-suite.org/meme/ (accessed on 20 May 2021)) online software to identify the occurrence of conserved motifs by employing the following parameters: normal mode, any number of repetitions, and two motifs. Analysis of the *MYB10* gene structure, including the intron and exons, was acquired from the *Fragaria* genome annotations available in rosaceae database (https://www.rosaceae.org (accessed on 20 May 2021)) and visualized using Gene Structure Server version 2.0 (http://gsds.cbi.pku.edu.cn/ (accessed on 20 May 2021)). For cis-element analysis, the 2 kb upstream transcriptional start site of each *MYB10* from diploids and octoploids was investigated using Plant Care (http://bioinformatics.psb.ugent.be/webtools/plantcare/html/ (accessed on 7 July 2021)) tool.

### 4.4. RNA-Seq Analysis of Skin and Flesh

Skin color of three cultivars ‘Mannyeonsul’, ‘Maehyang’, and ‘P69’ were measured by colorimeter (CR-400, Minolta, Japan) at mature stage. The skin and flesh tissues were separated from matured fruit samples using a scalpel according to Sánchez-Sevilla et al., [32]. Three biological replicates were employed for transcriptome analysis. The samples were homogenized and the total RNA was extracted using Trizol (Invitrogen, Waltham, MA, USA) according to the manufacturer’s protocol. After the quality evaluation, cDNA libraries were prepared from the RNA samples and paired-end library was constructed using the Truseq stranded mRNA Prep kit (Illumina Inc., San Diego, CA, USA) according to the manufacturer’s instructions. After purification, the sequencing library was produced by PCR amplification and sequenced using the Novaseq6000 platform (Illumina Inc., San Diego, CA, USA). The raw reads with low quality and the clean reads were then assembled and mapped to the ‘Wongyo 3115’ reference genome (Genebank accession number: JACXYW000000000) using the Top hat v2.0.13. The differential expression was analyzed using the cuffdiff v2.2.0. Genes with the FPKM estimate 2-fold higher than that of the lowest one were identified as differentially expressed genes (DEGs). Gene expression differences were validated using a chi-square test and false discovery rate (FDR). Genes with an FDR <0.001, and for which the FPKM estimate was 2-fold higher than that of the lowest one, were identified as DEGs. The functional annotations were performed using DAVID 6.8 Beta. A heat map was generated using significantly altered genes in fruits of both cultivars. The raw intensity data (FPKM) were log2 transformed and then utilized for the calculation of Z scores.

### 4.5. RT-PCR Analysis

The flesh tissues of mature fruits were separated from ‘Wongyo 3115’, ‘Mannyeonsul’, ‘Maehyang’, and ‘P69’. RNA isolation was performed in similar manner with transcriptome analysis as mentioned in the previous section. The cDNA biosynthesis was performed using a Quantitect Reverse Transcription Kit (Qiagen, Hilden, Germany) and RT-PCR was performed as described previously [35]. Gene expression level was calculated relatively to ‘Wongyo 3115’. All primers were designed based on the annotation data of ‘Wongyo 3115’ reference genome and are listed in Table S6.

### 4.6. QTL Analysis and Identification of Candidate Genes

The fruit color related traits of the ‘BS F_2_ (II)’ population were evaluated as two to three scales (Table S2). QTL analysis was performed using the phenotypes of 2019 and 2020 and genetic map constructed by Lee and Manivannan et al. [12]. Composite interval mapping of Windows QTL Cartographer 2.5 [36] with default option was used for QTL analysis. By using the 500 times permutation test (*p*-value < 0.05), significant QTLs exceeding the LOD threshold were selected. Based on the physical position of QTLs in ‘Wongyo 3115’ genome, overlapped QTLs were combined to one QTL and named based on the sub-chromosome number. For example, six QTLs were detected on chromosome 1-2 and combined to one QTL named ‘*Col_1-2*’ (Table 2). By comparison of QTL analysis and RNA-Seq results, candidate genes were selected. In detail, genes expressed differentially with respect to ‘Maehyang’ or ‘P69’ in comparison with ‘Mannyeonsul’ and located in the QTL region were considered as candidate genes for fruit color.

## Figures and Tables

**Table 1:** Characterization of *MYB10* transcription factor in *Fragaria.*

Species	Chr.	Gene ID	Location	Ploidy Level
*F. iinumae*	1	*evm.model.scaf_28.444*	14,574,366–14,576,255	Diploid
*F. nilgerensis*	1	*evm.model.ctg92.252_scbg_v1.0*	17,100,850–17,104,063	Diploid
*F. nubicola*	1	*evm.model.ctg173.372*	15,072,390–15,075,138	Diploid
*F. viridis*	1	*evm.model.ctg108.134*	13,877,866–13,880,523	Diploid
*F. vesca*	1	*FvH4_1g22020*	13,950,290–13,952,507	Diploid
*F.* × *ananassa* ‘Wongyo 3115’	1-1	*g00101712*	13,223,169–13,223,636	Octoploid
	1-2	*g00119641*	12,622,806–12,626,007	
	1-3	*g00115370*	13,537,353–13,539,011	
	1-4	*g00128511*	13,235,156–13,238,279	
*F.* × *ananassa* ‘Camarosa’	1-1	*maker-Fvb1-1-snap-gene-139.18*	13,899,904–13,911,034	Octoploid
	1-2	*maker-Fvb1-2-snap-gene-157.15*	15,722,359–15,726,641	
	1-3	*maker-Fvb1-3-augustus-gene-144.30*	14,468,714–14,470,589	
	1-3	*maker-Fvb1-3-augustus-gene-143.29*	14,315,382–14,315,713	

**Table 2:** QTLs for fruit color detected in the ‘BS F_2_ (II)’ population.

QTL	Chr.	Physical Position (Mbp)	Trait	Position (cM)	LOD	Additive Effect ^1^	Dominant Effect ^2^	R^2^ (%)
*Col_1-2*	1-2	12.1–24.5	Flesh_color (2020)	79.1–124.5	28.77	0.68	−0.35	75.94
			Achene_color (2020)	81.2–88.2	4.51	0.12	−0.45	29.78
			Core_color (2020)	82.2–142.9	32.02	0.41	0.30	38.48
			Achene_color (2020)	89.2–111.5	4.40	−0.17	−0.43	10.00
			Skin_color (2019)	97.5–142.9	7.19	0.23	0.18	12.91
			Skin_color (2020)	97.5–142.9	11.49	0.45	0.23	20.19
*Col_1-4*	1-4	11.8–15.9	Achene_color (2020)	53.4–66.6	5.67	−0.22	−0.10	10.04
*Col_2-1_1*	2-1	18.8–20.8	Flesh_color (2020)	74.5–100.4	4.45	0.91	−0.89	55.68
*Col_2-1_2*	2-1	21.2–26.9	Flesh_color (2020)	30.7–47.3	5.52	−0.28	−0.07	6.06
*Col_6-4_1*	6-4	3.0–6.9	Achene_color (2019)	7.0–17.9	3.97	−0.03	−0.25	5.67
*Col_6-4_2*	6-4	15.5–29.7	Skin_color (2019)	127.7–143.7	5.00	0.30	−0.25	0.62
			Achene_color (2020)	138.7–144.2	4.17	−0.26	0.11	15.09
*Col_7-4*	7-4	17.8–21.8	Achene_color (2020)	58.5–68.1	4.49	−0.18	−0.05	6.50

^1,2^ Positive and negative values mean that the genotype resembles that of maternal or paternal line, respectively.

**Table 3:** Predicted candidate genes in QTLs associated with the fruit color in the ‘BS F_2_ (II)’ population.

QTL	Gene ID	Chr.	Description	Function
*Col_6-4_1*	g00015421	6-4	DFR: Bifunctional dihydroflavonol 4-reductase/flavanone 4-reductase	Anthocyanin biosynthesis/Secondary metabolism
*Col_6-4_2*	g00017208	6-4	ANS1: Leucoanthocyanidin dioxygenase 1	Anthocyanin biosynthesis/Secondary metabolism
*Col_6-4_2*	g00018684	6-4	C1: Anthocyanin regulatory C1 protein	Anthocyanin biosynthesis
*Col_7-4*	g00137772	7-4	CHI3: Probable chalcone--flavonone isomerase 3	Anthocyanin biosynthesis/Secondary metabolism
*Col_7-4*	g00138590	7-4	GT1: Anthocyanidin 3-O-glucosyltransferase 1	Anthocyanin biosynthesis/Secondary metabolism
*Col_1-2*	g00119641	1-2	MYB113: Transcription factor MYB113/MYB10	DNA binding/Secondary metabolism
*Col_1-4*	g00128511	1-4	Similar to PNS1: Protein PNS1/MYB10	DNA binding/Secondary metabolism
*Col_7-4*	g00138251	7-4	UGT88B1: UDP-glycosyltransferase 88B1	
*Col_6-4_2*	g00018719	6-4	CYP750A1: Cytochrome P450 750A1	ABA biosynthesis
*Col_6-4_2*	g00019239	6-4	GT6: UDP-glucose flavonoid 3-O-glucosyltransferase 6	Anthocyanin biosynthesis/Secondary metabolism
*Col_6-4_2*	g00018681	6-4	GPI: Glucose-6-phosphate isomerase	Sugar metabolism

## Data Availability

Whole-genome sequence data of ‘Wongyo 3115’ have been deposited in NCBI under the Bioproject PRJNA662854 and Biosample SAMN16094694 (accession number SRR14102268-SRR14102276). This whole genome shotgun project has been deposited at GenBank under the accession JACXYW000000000. The version described in this paper is JACXYW010000000.

## Supplementary Materials

The following are available online at https://www.mdpi.com/article/10.3390/ijms222212587/s1.
